# Prognostic value of TERF1 expression in prostate cancer

**DOI:** 10.1186/s43046-021-00082-4

**Published:** 2021-09-06

**Authors:** Gabriel Arantes dos Santos, Nayara Izabel Viana, Ruan Pimenta, Vanessa Ribeiro Guimarães, Juliana Alves de Camargo, Poliana Romão, Sabrina T. Reis, Katia Ramos Moreira Leite, Miguel Srougi

**Affiliations:** 1 Urology Department, Laboratory of Medical Investigation (LIM55), Faculdade de Medicina FMUSP, Universidade de Sao Paulo, Room 2145 01246-903, 2° Floor, Av. Dr. Arnaldo 455, Sao Paulo, SP Brazil; 2 D’Or Institute for Research and Education (IDOR), Sao Paulo, Brazil; 3 Minas Gerais State University (UEMG), Passos, Minas Gerais Brazil

**Keywords:** Prostate cancer prognosis, TCGA, Telomere, Shelterin

## Abstract

**Background:**

Telomere dysfunction is one of the hallmarks of cancer and is crucial to prostate carcinogenesis. TERF1 is a gene essential to telomere maintenance, and its dysfunction has already been associates with several cancers. TERF1 is a target of miR-155, and this microRNA can inhibit its expression and promotes carcinogenesis in breast cancer. We aim to analyze TERF1, in gene and mRNA level, involvement in prostate cancer progression.

**Results:**

Alterations in TERF1 DNA were evaluated using datasets of primary tumor and castration-resistant tumors (CRPC) deposited in cBioportal. The expression of TERF1 mRNA levels was assessed utilizing TCGA datasets, clinical specimens, and metastatic prostate cancer cell lines (LNCaP, DU145, and PC3). Six percent of localized prostate cancer presents alterations in TERF1 (the majority of that was amplifications). In the CRPC cohort, 26% of samples had TERF1 amplification. Patients with TERF1 alterations had the worst overall survival only on localized cancer cohort (*p* = 0.0027). In the TCGA cohort, mRNA levels of TERF1 were downregulated in comparison with normal tissue (*p* = 0.0013) and upregulated in tumors that invade lymph nodes (*p* = 0.0059). The upregulation of TERF1 is also associated with worst overall survival (*p* = 0.0028) and disease-free survival (*p* = 0.0023). There is a positive correlation between TERF1 and androgen receptor expression in cancer tissue (*r* = 0.53, *p* < 0.00001) but not on normal tissue (*r* = − 0.16, *p* = 0.12). In the clinical specimens, there is no detectable expression of TERF1 and upregulation of miR-155 (*p* = 0.0348). In cell lines, TERF1 expression was higher in LNCaP and was progressively lower in DU145 and PC3 (*p* = 0.0327) with no differences in miR-155 expression.

**Conclusion:**

Amplification/upregulation of TERF1 was associated with the worst prognostic in localized prostate cancer. Our results corroborate that miR-155 regulates TERF1 expression in prostate cancer. TERF1 has the potential to become a biomarker in prostate cancer.

## Background

Telomeres are the structural ends of eukaryotic chromosomes and are formed by tandem repeats of the 5′-TTAGGG-3′ sequence in mammals. They control the number of cell divisions and maintain genomic stability [1].

Many proteins highly regulate the telomere structure, the most important being a protein complex called shelterin. Shelterin is a multiprotein complex composed of six subunits (TRF1, TRF2, POT1, TPP1, TIN2, and RAP1) that binds to the telomere, protecting, and regulating telomere length [2].

Telomere repeat-binding factor 1 (TRF1) is translated from the TERF1 gene (8q21.11) and directly binds to the telomere, acting as a protector of telomeres and a negative regulator of telomerase activity, the enzyme responsible for telomere elongation and cellular immortalization in 85 ~ 90% of all cancers. Among TERF1 functions, we highlight its recruitment of and interaction with PINX1 to inhibit telomerase activity. PINX1 was previously shown to be downregulated in PC and related to cellular immortalization [3].

Telomeric dysfunction and abnormal expression of telomeric components are reported in most cancers [4]. In prostate cancer (PC), reactivation of telomerase and severe telomere shortening are considered primary steps in the disease, and both phenotypes may be related to TERF1 functions [5].

MicroRNAs are small molecules that control gene expression at the post-transcriptional level and have been related to many dysfunctions during carcinogenesis, acting as oncogenes or tumor suppressor genes [6].

MiR-155 is upregulated in breast cancer and acts as a key regulator of the shelterin component TRF1 by targeting a conserved sequence motif in the 3′UTR of TERF1 [6]. In the context of breast carcinogenesis, downregulation of TERF1 promotes genetic instability and facilitates cell immortalization, being a key event in cancer initiation. Besides that, TERF1 plays a crucial role in several neoplasms.

In this work, we analyzed the involvement of TERF1 in PC progression utilizing public datasets, clinical specimens, and the three most important PC metastatic cell lines.

## Methods

### DNA alterations in prostate cancer datasets

The genomic alterations of TERF1 in the PC were evaluated using The Cancer Genome Atlas (TCGA) (*n* = 499) and a SU2C/PCF cohort (*n* = 444) datasets deposited in the public repository cBioPortal [7–9].

The TCGA cohort was composed of primary tumor samples and the SU2C/PCF cohort by metastatic castration-resistant prostate cancer samples.

All images are generated from cBioPortal with minor styles adjustments.

### TERF1 expression on TCGA prostate cancer datasets

We use data from mRNA expression levels of TCGA PC datasets.

All analyses were made using the online UALCAN and GEPIA (Gene Expression Profiling Interactive Analysis) database [10, 11]. The TCGA population was composed of 497 tumor samples, and 52 paired normal samples. In Kaplan–Meier plots, the expression data were normalized by B2M expression.

All images are generated from UALCAN or GEPIA, with minor styles adjustments.

### Patients

This study was submitted and approved by the Research Ethics Committee of the Medical School of the University of São Paulo under the number 3,196,078. All participants signed the Informed Consent Form and were informed about safety in terms of integrity.

Fifty tissue samples obtained from radical prostatectomy specimens were the subject of the study. The clinical characteristics of the patients from which the samples were taken are shown in Table 1. The control group consisted of 10 tissue samples obtained from benign prostate hyperplasia (BPH) specimens. None of the patients developed metastasis.

**Table 1:** Patient characteristics

		Gleason (***n***)			Cancer stage (***n***)	
PSA (ng/ml)	Age (years)	6	7	8 or 9	pT2	pT3
8.06 (± 10.53)	59.44 (± 8.26)	24	12	14	40	10

### Cell lines

The cell lines DU145, PC3, and LNCaP were obtained from the American Type Culture Collection (Manassas, VA, USA) and cultured using MEM (DU145 and PC3) and RPMI (LNCaP) medium (Life Technologies, Waltham, MA, USA) supplemented with 10% fetal bovine serum and 1% antibiotic/antimycotic solution (Sigma Co., St. Louis, MO, USA) at 37 °C in an atmosphere of 5% CO2. The cell lines were authenticated by STR profile. The gene expression assays were performed in triplicate.

### TERF1 and miR-155 expression analyses

A MirVana kit (Ambion, Austin, TX, USA) was used for RNA and miRNA extraction; cDNA was obtained using the TaqMan miRNA Reverse Transcription kit (Applied Biosystems, Foster City, CA, USA) according to the manufacturer’s recommendations.

The RNA and microRNA expression levels were analyzed by Q-PCR using the ABI 7500 Fast Real-Time PCR System (Applied Biosystems). The target sequences were amplified in a 10-μL reaction containing 5-μL TaqMan Universal PCR Master Mix, 0.5 μL TaqMan Gene Expression Assays (TERF1-Hs00819517_mH; and miR-155-002623), 1 μL cDNA, and 3.5 μL DNase-free water. The PCR cycling conditions were as follows: 2 min at 50 °C, 10 min at 95 °C, and 45 cycles of 15 s at 95 °C and 1 min at 60 °C. All reactions were performed in duplicates. TaqMan B2M (Hs_00187842_m1) and RNU 48 (001006) were utilized as endogenous controls for gene and miRNA expression.

### Analysis of results

For bioinformatics data, statistical analyses were performed automatically by the software used (cBioPortal, GEPIA, or UALCAN). To compare genetic alteration between the two bioinformatic cohorts, we used the Fisher exact test. Ordinary one-way ANOVA was performed for hypothesis test in the association between mRNA levels and CNV status. In Kaplan–Meier plots, the hypothesis test used was the Log-rank test (Mantel-Cox test). In boxplots expression analysis, the *p* value was obtained by Student’s *t* test (considering unequal variance). The correlation coefficient was assessed using Pearson’s test.

For experimental data, gene expression was calculated using DataAssist v3.01 software. The graphs were generated, and the statistical analysis was performed using GraphPad Prism 8 software. To compare gene and microRNA expression, we used Welch’s *t* test for two groups and one-way ANOVA with Bonferroni’s correction for three groups. We set a level of significance of 5% (*p* < 0.05).

## Results

### TERF1 amplification is associated with worst prognostic on primary prostate cancer

**Fig. 1:**
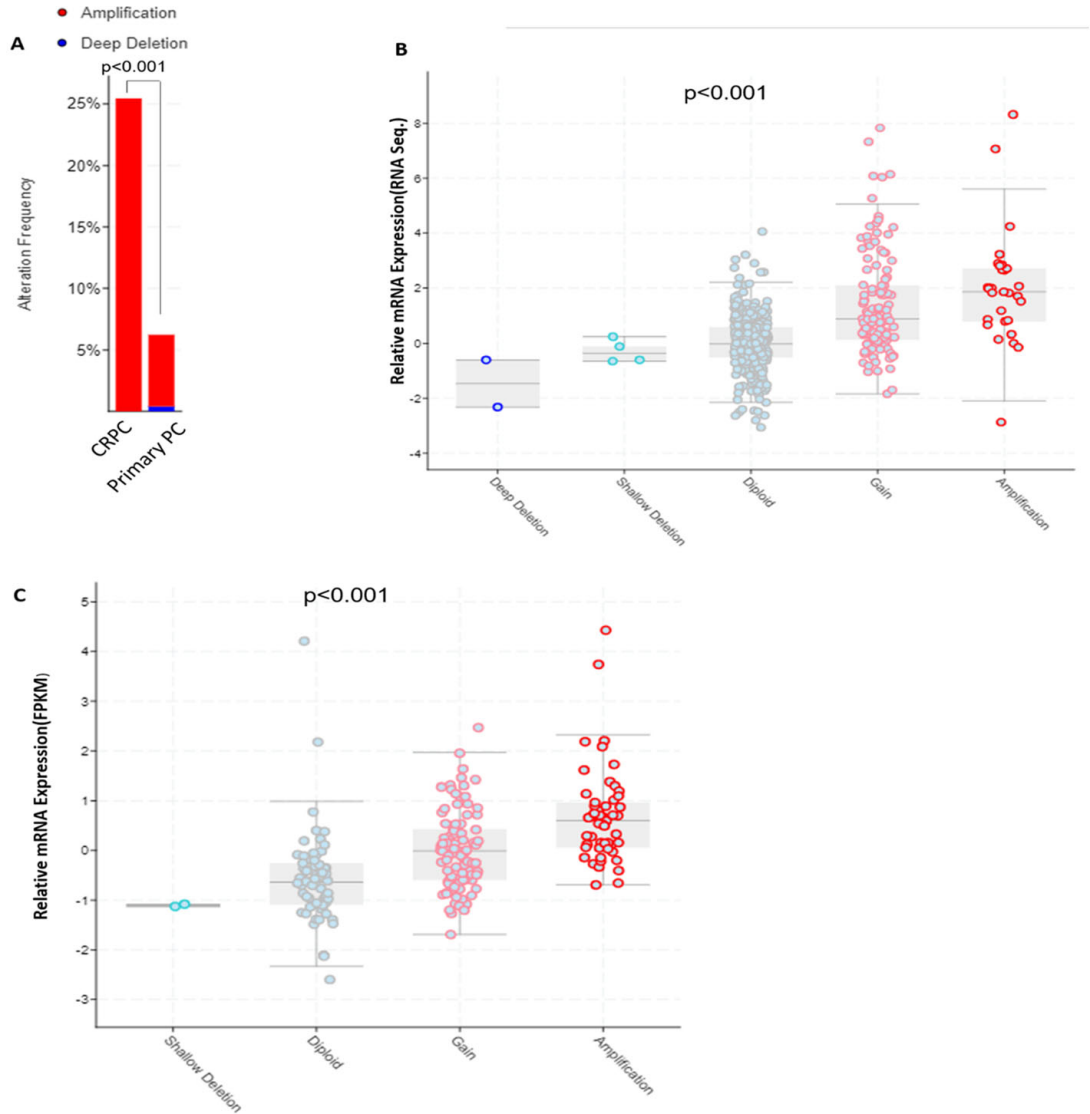
Comparison of TERF1 in primary tumor and CRPC cohort. **A** Gene amplification in both primary and CRPC cohorts. **B** TERF1 gene expression in primary tumor. **C** TERF1 gene expression in CRPC due to gene amplification

First, we analyzed the alterations on TERF1 DNA in PC, considering both mutations and copy number variations (CNV); these results were summarized in Fig. 1A. No mutations were identified in primary cancer or castration-resistant prostate cancer (CRPC) cohorts. In the primary cancer cohort, 6% of patients had alterations in TERF1, and vast majority of these were amplification. In the CRPC cohort, 26% (about 1 in 4) of the patients had TERF1 amplifications. In addition, the gain/amplification of this gene increased the expression levels of TERF1 (Fig. 1B, C).

**Fig. 2:**
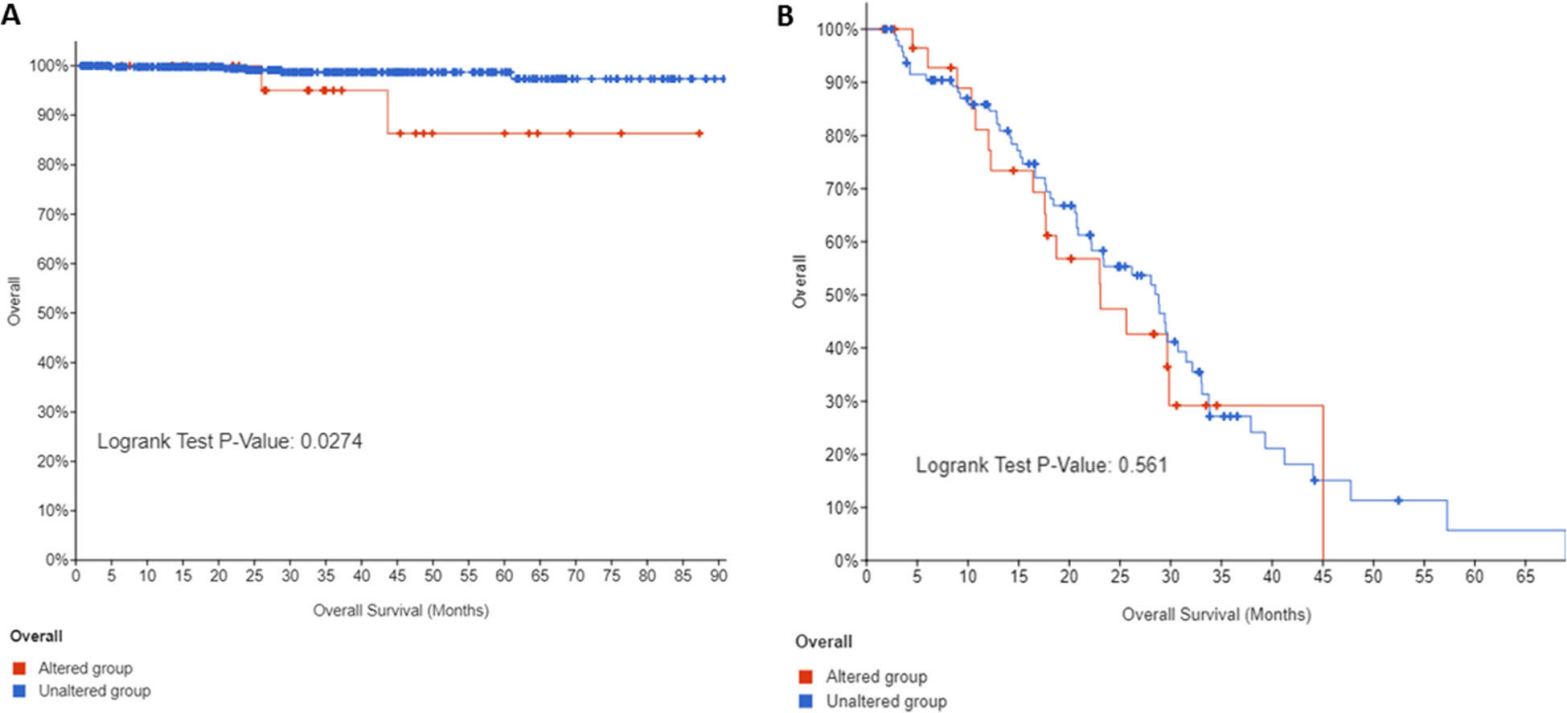
Overall survival of patients with or without alterations in TERF1. **A** Kaplan–Meier plot from primary tumor. **B** Kaplan–Meier plot from castration resistant metastatic tumors

In primary cancer, patients without alterations in TERF1 had a better overall survival (Fig. 2A, *p* = 0.027), but in the CRPC cohort the alterations did not change patient survival (Fig. 2B, *p* = 0.56).

### Expression of TERF1 upregulation is associated with PC aggressiveness on TCGA datasets

Our previous results suggest that upregulation of TERF1 may predict which primary tumor will become an aggressive PC. This can be extremely relevant in the context of this PC, since overdiagnosis and overtreatment are a challenge, exacerbating the need for new molecular biomarkers that could stratify which patients need invasive treatment (for having an aggressive cancer) and which patients would have an indolent disease [12].

**Fig. 3:**
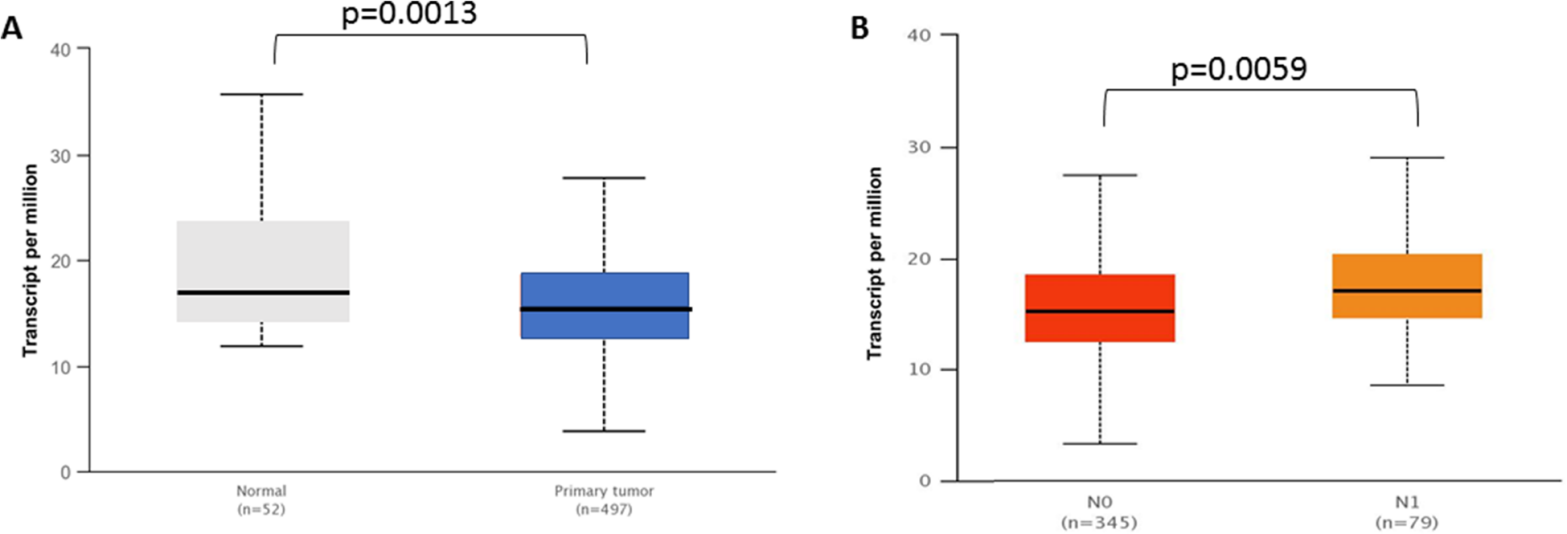
Gene expression analysis of TERF1 in TCGA cohort. **A**. Comparison between normal tissue and prostate cancer. **B** Comparison between tumors that did not invade proximal lymph nodes (N0) and tumors that invade proximal lymph nodes (N1)

To test this hypothesis, we analyzed the TERF1 expression on primary PC utilizing two different platforms. First, TERF1 is downregulated in cancer when compared with normal tissue (Fig. 3A, *p* = 0.0013).

**Fig. 4:**
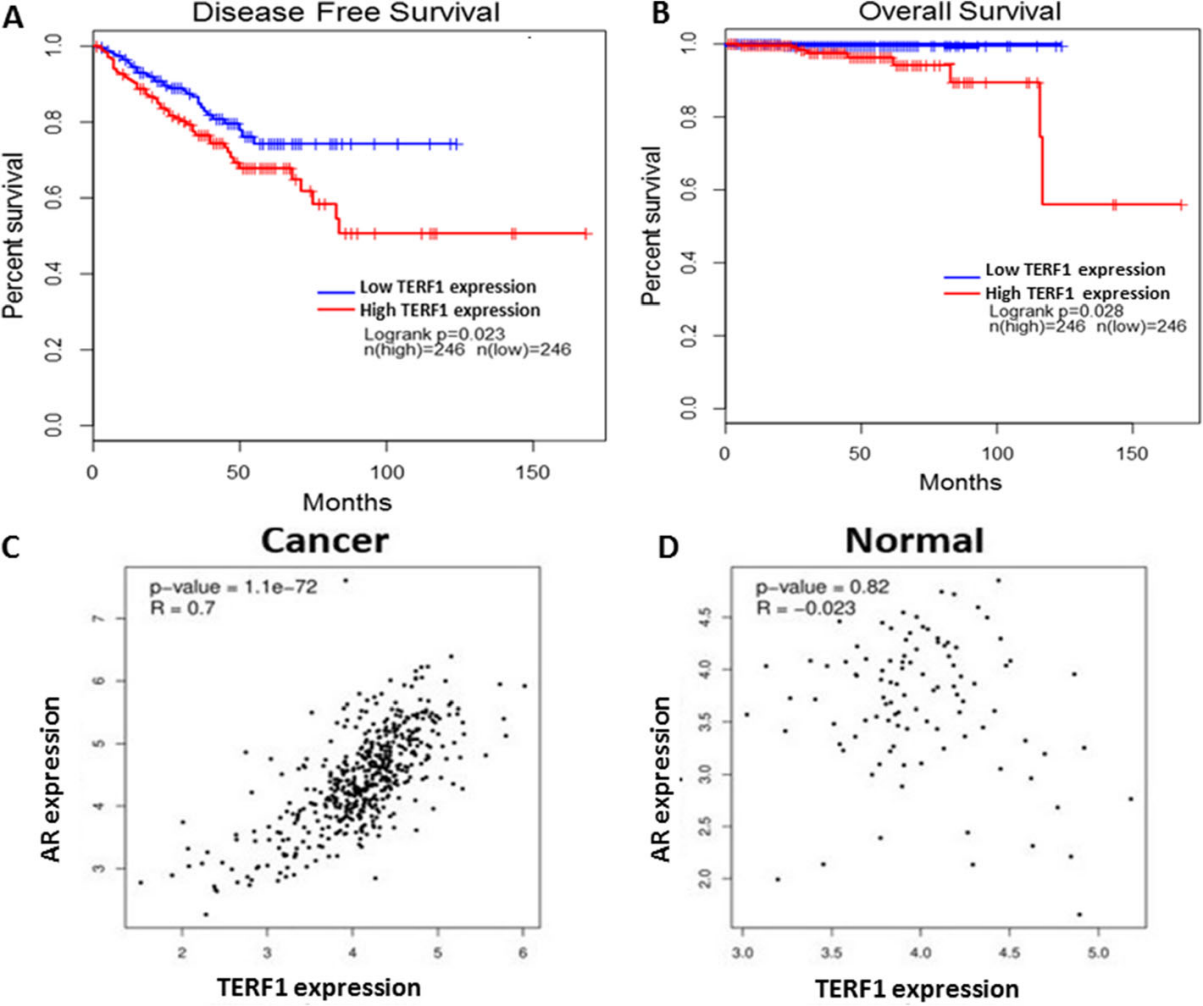
Association between TERF1 gene expression with prognosis. **A** Kaplan–Meier disease-free survival plot. **B** Kaplan–Meier overall survival plot. **C** Correlation of AR expression with TERF1 in cancer. **D** Correlation of AR expression with TERF1 in normal tissue

Next, we observed that tumors that invade local lymph nodes present TERF1 upregulation (Fig. 3B, *p* = 0.0059). A recent work, utilizing the same software (UALCAN), made similar observations and shows that TERF1 upregulation were more common in cancers with higher Gleason scores [13]. TERF1 upregulation was also associated with poor disease-free survival (Fig. 4A, *p* = 0.023) and overall survival (Fig. 4B, *p* = 0.028).

Finally, we correlated TERF1 expression with androgen receptor (AR) expression. AR was chosen because of its association with progression of PC and telomere maintenance in cancer [14–16]. TERF1 expression had a strong positive correlation with AR on cancer tissue (Fig. 4C, *r* = 0.53, *p* < 0.00001) but not on normal tissue (Fig. 4D, *r* = − 0.16, *p* = 0.12), again suggesting a better prognostic associated with low TERF1 expression.

### Upregulation of miR-155 is associated with TERF1 depletion in PC clinical samples.

Since the downregulation of TERF1 may be related to a better prognosis in PC, we collected surgical specimens from radical prostatectomy and evaluated the levels of TERF1 mRNA. It is important to note that, despite a large variation in classic PC prognostic markers (PSA, Gleason score and cancer stage, showed in Table 1), all of these patients had a good clinical evolution, without developing metastases or cancer-specific death. We choose this cohort because classical prognostic factors are not accurate enough to separate indolent from aggressive cancers in a totally reliable way.

**Fig. 5:**
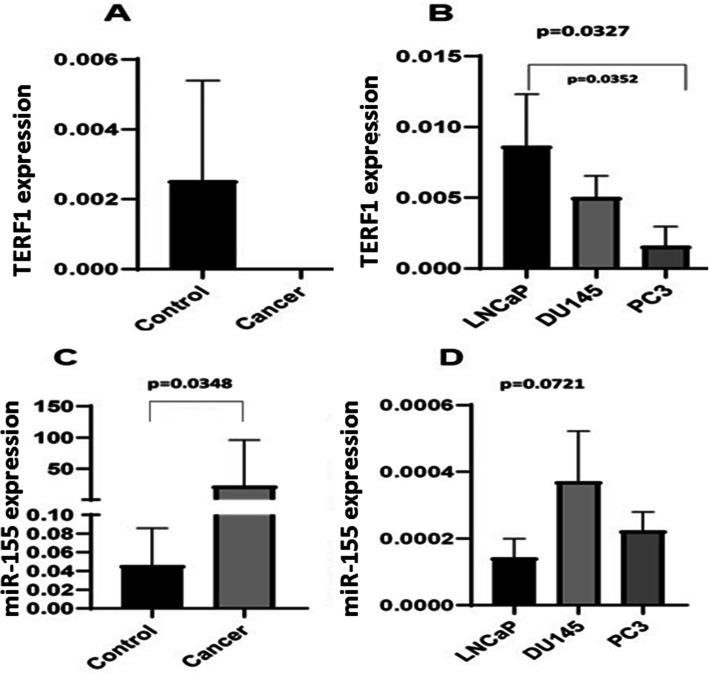
Upregulation of miR-155 is associated with TERF1 depletion in PC clinical samples and cell lines. **A** TERF1 gene expression in PC surgical specimens. **B** TERF1 expression in PC cell lines. **C** miR-155 expression in surgical specimens. **D** miR-155 expression in PC cell lines. The graph represents the mean expression in the population with the standard deviation. The *y* axis is in arbitrary unit

Surprisingly, we did not detect expression of TERF1 in any of our samples (Fig. 5A). As miR-155 is the only microRNA identified in the literature as an inhibitor of TERF1, and has already been associated with deregulation of this gene in breast and prostate cancer samples, we verified its expression [6, 13]. MiR-155 was highly upregulated in the clinical specimens compared with the control group (Fig. 5C, *P* = 0.0358), which could explain the absence of TERF1 expression.

### TERF1 involvement in PC progression

To further investigate the role of TERF1 in PC, we analyzed TERF1 and miR-155 expression in three metastatic PC cell lines. There was a progressive downregulation of TERF1 from LNCaP (derived from lymph node metastasis of a castration-sensitive PC) to DU145 (derived from brain metastasis of CRPC) and PC3 (derived from bone metastasis of CRPC) (*p* = 0.0327) (Fig. 5B). There was also a significant difference in TERF1 expression between LNCaP and PC3 cells (*p* = 0.0352).

All three cell lines show detectable expression of TERF1 but, the miR-155 expression cannot explain the difference between the cell lines (Fig. 5D, *p* = 0.072), suggesting another mechanism of control.

## Discussion

Telomere dysfunction is one of the hallmarks of cancer and is related to shelterin dysfunction. TRF1 is a protein that binds directly to double-stranded telomeric DNA, interacting directly or indirectly with other shelterin components, telomerase, DNA repair machinery, and other proteins responsible for telomere maintenance [2]. The loss of TERF1 is related to genomic instability, a characteristic related to PC development and progression [5, 6]. It is interesting to note that TERF1 is in the 8q.21 region and amplifications in that region of the chromosome have already been associated with a worse prognosis for PC [17]. In the present study, we tried to understand the TERF1 role in PC.

First, we observed a great increase in the number of amplifications in TERF1 on CRPC. These results may lead us to conclude that TERF1 amplification is an important event in the acquisition of the lethal phenotype in PC, where cancer evolves to metastasis resistant to treatments. Since normally, the patients with PC only die when the disease progress to CRPC, we next check whether alterations on TERF1 can impact the overall survival of these patients [18].

The survival analysis suggests that amplifications in TERF1 are associated with cancer aggressiveness only on the primary PC. The probable reason for the large proportion of alterations in the CRPC is due because it is exactly these more aggressive tumors that evolve to the lethal phenotype, but the amplification itself does not change the prognosis for these patients.

Then, we demonstrate a downregulation of TERF1 in both TCGA and clinical cohorts of localized PC. This phenomenon is expected since the downregulation of TERF1 can leads to increase in genetic instability and may favor the cellular immortalization, remarkable features at the onset of prostate carcinogenesis, with similar observations observed in breast cancer [5, 6].

On the contrary, the upregulation of TERF1 may be related with poor prognosis on primary PC, being associated with poor overall and disease-free survival, lymph node invasion, and AR upregulation.

Besides that, we showed a correlation between TERF1 and AR only on cancer tissue. This is relevant because studies showed that androgen axis modulates telomere length in normal cells, which suggest that AR control telomeres [19]. Specifically on prostate, it is postulated that AR repress the expression of telomerase in normal cells, but reactivates the enzyme in cancer cells, which favors cancer initiation and progression [5]. In addition, AR interacts directly with shelterin through TIN2 (which binds directly to TRF1) and AR antagonist promotes telomere dysfunction only in AR positive PC cell lines [5, 16]. Our result reinforce that AR modulates telomere dynamics during prostate cancer progression, and, maybe TERF1 plays a crucial role in this process.

In the clinical cohort, we corroborate that miR-155 overexpression can causes the depletion of TERF1. MiR-155 is a well-known oncomiR and has been shown to be overexpressed in many cancers, including PC [20]. Despite the extensive literature about this microRNA, it is most commonly associated with the regulation of genes related to inflammation, the immune system and hematopoiesis [20]. In this study, we showed that miR-155 is upregulated in primary PC samples that do not show any detectable expression of TERF1.

Despite the importance of microRNAs in several biological processes, the relationship between them and telomeres is not well studied. For example, in addition to TERF1 and miR-155, only three others microRNAs have already been validated by regulating other shelterin genes (miR-490 and miR-23a for TERF2 and miR-185 for POT1) [21–23]. In addition, there are studies that show that telomerase is regulated by several microRNAs, which indicates that the interaction between telomeres and microRNAs is a vast field that should be more explored in future studies.

Considering metastatic PC, our results support the possibility that TERF1 expression is important for telomere maintenance and cancer cell immortalization in advanced cancer stages, by protecting the cell of telomeric DNA damage and promoting cancer cell stemness.

The induction of telomere uncapping by TERF1 genetic depletion has been shown to effectively block the initiation and progression of aggressive tumors in lung and glioblastoma mouse models, and some studies have recently proposed the induction of TERF1 depletion as a possible molecular treatment in glioblastoma multiforme [24, 25].

On the contrary, during tumor progression to advanced stages, telomere shortening, chromosome instability, and increased tumor aggressiveness had been related to progressive downregulation of TERF1. The promotion of cancer aggressiveness by TERF1 downregulation already been reported in metastatic PC models [13]. Our results using metastatic cell lines reinforce this theory. In the three most important PC cell lines, we were able to show that there is a progressive downregulation of TERF1 from a castration-sensitive cell line to the castration-resistant cell lines. In breast cancer, TERF1 downregulation is related to higher genomic instability and an increase in radioresistance [6, 26]. In PC, there has only been one study showing that overexpression of TERF1 is related to unfavorable prognostic factors [27]. Here, we also showed that low TERF1 expression was related to a better prognostic on localized PC.

We advocate that TERF1 depletion and telomeric dysfunction are important for the first steps of carcinogenesis (localized PC) and are supplanted by other dysfunctions during tumor progression.

After that, probably to avoid genetic crisis, some cancer cells recover TERF1 expression, probably by an AR-dependent mechanism, and progress to an aggressive phenotype with a certain level of genomic stability (such as seen in LNCaP cells and advanced localized tumors). Here, it is important to remember that, even though genetic instability is a hallmark of the cancer cell, very high levels of genomic instability can be harmful to cancer progression for several reasons (such as the increase of neoantigens).

Suggesting a mechanism, we postulate that overexpression of miR-155 suppresses TERF1 expression in the carcinogenesis process. This would favor genetic instability and cell immortalization promoting cancer initiation. After that, cells with AR upregulation increase the expression of TERF1, suppressing the excess of DNA damage at telomeres. Considering that TERF1 cannot inhibit telomerase activity (a tumor suppressor effect) without PINX1 (and that this protein is downregulated in advanced stages of the PC), this phenomenon can promote the evolution to a more aggressive stage of the cancer [3]. It is interesting to note that a recent study reports that TERF2 modulates PC progression by regulating telomere DNA damage pathways, which reinforce the involvement of shelterin in disease progression [28].

Amplifications of TERF1 were enriched in CRPC datasets, but these alterations were not too relevant in this stage of the cancer. Another important characteristic of CRPC is that AR amplification/reactivation is very common. In DU145 and PC3 (CRPC cell lines), we showed a downregulation of TERF1 which is coherent. In these two cell lines, the downregulation of TERF1 is probably associated with the resume of genetic instability, present in both cells, and the fact that they are AR-negative.

## Conclusion

In summary, we identified that amplification/upregulation of TERF1 may be linked to a population of more aggressive PC cells and may have an important role in disease progression, having the potential to become a biomarker. Our work also corroborates that miR-155 can target TERF1 and be important to prostate carcinogenesis.

## Data Availability

The datasets used and/or analyzed during the current study are available from the corresponding author upon reasonable request.

## Abbreviations

3′UTR: Three prime untranslated region
AR: Androgen receptor
BPH: Benign prostate hyperplasia
CNV: Copy number variation
CRPC: Castration-resistant prostate cancer
GEPIA: Gene Expression Profiling Interactive Analysis
MiR-155: MicroRNA 155
PC: Prostate cancer
PCR: Polymerase chain reaction
Q-PCR: Quantitative polymerase chain reaction
TCGA: The Cancer Genome Atlas
TERF1: Telomere repeat-binding factor 1
